# ASPECTS-based selection for late endovascular treatment: a retrospective two-site cohort study

**DOI:** 10.1177/17474930211009806

**Published:** 2021-04-22

**Authors:** Stefania Nannoni, Johannes Kaesmacher, Federico Ricciardi, Davide Strambo, Vincent Dunet, Steven Hajdu, Guillaume Saliou, Pasquale Mordasini, Arsany Hakim, Marcel Arnold, Jan Gralla, Urs Fischer, Patrik Michel

**Affiliations:** 1 Stroke Center, Neurology Service, 30635 Lausanne University Hospital and University of Lausanne, Lausanne, Switzerland; 2 Institute of Diagnostic and Interventional Neuroradiology, University Hospital Bern, Inselspital, Bern, Switzerland; 3 Institute of Diagnostic, Interventional and Pediatric Radiology, University Hospital Bern, Inselspital, Bern, Switzerland; 4 Department of Diagnostic and Interventional Radiology, 30635 Lausanne University Hospital, and University of Lausanne, Lausanne, Switzerland; 5 Department of Neurology, Inselspital, Bern University Hospital, University of Bern, Bern, Switzerland

**Keywords:** Acute ischemic stroke, late time window, endovascular treatment, ASPECTS

## Abstract

**Introduction:**

The DAWN trial demonstrated the effectiveness of late endovascular treatment in acute ischemic stroke patients selected on the basis of a clinical-core mismatch. We explored in a real-world sample of endovascular treatment patients if a clinical-ASPECTS (Alberta Stroke Program Early CT Score) mismatch was associated with an outcome benefit after late endovascular treatment.

**Methods:**

We retrospectively analyzed all consecutive acute ischemic stroke patients admitted 6–24 h after last proof of good health in two stroke centers, with initial National Institutes of Health Stroke Scale (NIHSS) ≥10 and an internal carotid artery or M1 occlusion. We defined clinical-ASPECTS mismatch as NIHSS ≥ 10 and ASPECTS ≥ 7, or NIHSS ≥ 20 and ASPECTS ≥ 5. We assessed the interaction between the presence of the clinical-ASPECTS mismatch and late endovascular treatment using ordinal shift analysis of the three-month modified Rankin Scale and adjusting for multiple confounders.

**Results:**

The included 337 patients had a median age of 73 years (IQR = 61–82), admission NIHSS of 18 (15–22), and baseline ASPECTS of 7 (5–9). Out of 196 (58.2%) patients showing clinical-ASPECTS mismatch, 146 (74.5%) underwent late endovascular treatment. Among 141 (41.8%) mismatch negative patients, late endovascular treatment was performed in 72 (51.1%) patients. In the adjusted analysis, late endovascular treatment was significantly associated with a better outcome in the presence of clinical-ASPECTS mismatch (adjusted odd ratio, aOR = 2.83; 95% confidence interval, CI: 1.48–5.58) but not in its absence (aOR = 1.32; 95%CI: 0.61–2.84). The *p*-value for the interaction term between clinical-ASPECTS mismatch and late endovascular treatment was 0.073.

**Conclusions:**

In our retrospective two-site analysis, late endovascular treatment seemed effective in the presence of a clinical-ASPECTS mismatch, but not in its absence. If confirmed in randomized trials, this finding could support the use of an ASPECTS-based selection for late endovascular treatment decisions, obviating the need for advanced imaging.

## Introduction

Recent randomized clinical trials have provided class I evidence for the efficacy of endovascular treatment (EVT) in acute ischemic stroke (AIS) patients from proximal anterior circulation large vessel occlusion (LVO) in the late-time window, if properly selected based on their neuroimaging profile.^1–3^ However, we previously demonstrated that the proportion of late-admitted AIS eligible for EVT according to strict trial criteria was low in the real-life scenario.^4^

Enlarging the selection criteria for late EVT could allow a larger population of AIS patients to benefit from the revascularization procedures. Notably, the use of a simpler neuroimaging protocol could help with the decision to proceed with mechanical thrombectomy in case of absent, failed or contraindicated advanced imaging, or in situations of discordant imaging profile.^5^

The Alberta Stroke Program Early CT Score (ASPECTS) is an easily applicable tool to estimate the amount of irreversibly damaged brain tissue in the middle cerebral artery (MCA) territory strokes.^6^ Originally designed for non-contrast CT scan (NCCT), it has been also applied to diffusion-weighted imaging (DWI) sequences, after one-point adjustment.^7^ However, the role of ASPECTS in selecting patients who are most likely to benefit from EVT is not clearly established in the late time window.^8,9^ Also, to the best of our knowledge, its use in association of clinical stroke severity as a surrogate of the core-penumbra mismatch^1^ has not been evaluated.

The main aim of our study was to analyze the clinical outcome of late-arriving AIS patients with proximal anterior circulation LVO depending on the presence of a clinical-ASPECTS mismatch and of treatment with mechanical thrombectomy in two comprehensive stroke centers.

## Methods

### Study design and study population

We performed a retrospective analysis of all consecutive AIS patients who received mechanical thrombectomy in the late time window in the comprehensive stroke centers of Lausanne and Berne University Hospitals from January 2010 to December 2018. Data were extracted from the prospectively constructed stroke registry of each institution, whose details have been previously published.^10,11^

For the current analysis, we adopted the following inclusion criteria: anterior circulation stroke with proximal LVO (i.e. occlusion of intracranial internal carotid artery (ICA), and/or M1 segment of the MCA); National Institutes of Health Stroke Scale (NIHSS) on admission ≥10; mechanical thrombectomy started between 6 and 24 h since last proof of good health (LPGH); and availability of three-month functional status, assessed with the modified Rankin scale (mRS).

For the current analysis, we defined a “clinical-ASPECTS mismatch” as the presence of NIHSS ≥10 combined with ASPECTS ≥7 or NIHSS ≥20 associated with ASPECTS ≥5. The ASPECTS was visually scored on the first unenhanced neuroimaging modality (NCCT or diffusion-weighted magnetic resonance imaging, DWI) obtained on admission. The clinical-ASPECTS definition was inspired by the clinical-core mismatch used in the DAWN trial,^1^ and based on the ASPECTS/CTP-core correlation that we previously investigated.^12^ In order to adjust for the higher sensitivity of DWI, one point was added before analysis for DWI-ASPECTS.^7^ Unlike the DAWN trial, we did not apply any age or pre-stroke functional disability cut-off, in order to present a closer picture to the real-life scenario. We therefore identified a group of treated patients presenting the clinical-ASPECTS mismatch (“mismatch positive-EVT”), and a group of treated patients with ASPECTS <5 or not showing the clinical-ASPECTS mismatch (“mismatch negative-EVT”).

As control group, we selected all consecutive AIS admitted to the Lausanne University Hospital in the late time window, and *not* receiving EVT (intravenous thrombolysis, IVT, allowed). The inclusion period for this group was extended from 2005 to 2018 to ensure a satisfactory sample size. The same clinical (i.e. NIHSS ≥10) and radiological (ICA or M1 occlusion) inclusion criteria were applied. Similarly, we identified two groups of non-treated patients: the “mismatch positive-no EVT” group, corresponding to non-treated patients showing the clinical-ASPECTS mismatch, and “mismatch negative -no EVT” group, i.e. non treated patients with ASPECTS <5 or without the clinical-ASPECTS mismatch.

Details on the neuroimaging protocol of each participating center, ASPECTS scoring and adopted EVT guidelines have been published before,^12,13^ and additional information are provided in the Supplementary Material.

The primary outcome of the study was the shift towards better functional outcome in the three-month mRS in the “mismatch positive-EVT” group compared to the “mismatch positive-no EVT” group, and in the “mismatch positive-EVT” vs. “mismatch negative-EVT” groups. As secondary outcomes, we chose the improvement of the NIHSS at 24 h from the baseline (delta NIHSS), and the rate of symptomatic intracerebral hemorrhage (sICH) defined according to the ECASS II criteria.^14^

### Statistics

We first analyzed demographic, clinical, and radiological variables from the acute phase of stroke, performing comparisons between the four study groups defined by the presence of clinical-ASPECTS mismatch (mismatch positive, mismatch negative) and acute treatment modality (late EVT, no EVT). Baseline characteristics of the cohort were summarized both overall and separately for each group of patients, reporting frequencies and percentages for binary and categorical variables, and medians (and inter-quartile ranges, IQR) for continuous measures. Comparisons were conducted using appropriate statistical testing, i.e. Mann–Whitney U for continuous variables and Chi-squared or Fisher Exact test for categorical variables.

The primary outcome was analyzed using both univariate and multivariable methods. We fitted ordered logit regression models, including interaction terms between the variables “clinical-ASPECTS mismatch” and “late EVT”, with the response variable being the shift towards favorable outcome (i.e. lower mRS) at three months. A likelihood ratio test was used to assess the assumption of proportional odds: no evidence of non-proportional odds has emerged across all the categories of mRS for each covariate in the model (*p* > 0.05 for all).

We obtained four different odds ratios (ORs) comparing the four different groups of patients, and the OR for the interaction term. First, unadjusted ORs were calculated, and then a multivariable analysis was performed, in which a list of variables was used to adjust the ORs of interest. These included variables that had been shown to influence outcome or because their unbalanced distribution among the four groups. The final, adjusted, model included the followings: age, pre-stroke mRS < 3, LPGH to hospital arrival time, admission NIHSS, admission glucose level, baseline ASPECTS, and IVT.

Also, a sensitivity analysis was performed in the subgroup of patients who had the CT as initial imaging modality.

Secondary outcomes were analyzed using comparative univariate logistic or linear regression models (depending on the nature of the outcome variable). Adjusted analyses were not performed for secondary outcomes.

Given the retrospective registry-based analysis of data, a formal sample size calculation was not performed. However, a post hoc sample size assessment was conducted: assuming an absolute benefit between patients receiving late EVT and non-treated patients being half of the one reported in the DAWN trial (i.e. 18%), we calculated that a sample size of 115 patients would be required in each group, considering 80% power and 5% alpha.

We performed complete case analyses, with only observations with complete information being considered; hence, no formal treatment of missing data was done.

Statistical analyses were performed using STATA version 15 and R version 4.0.

This study was approved by the hospital’s Institutional Review Board of each center for retrospective data collection and review.

### Data accessibility statement

Anonymized data will be shared by request from any qualified investigator.

## Results

**Figure 1.**
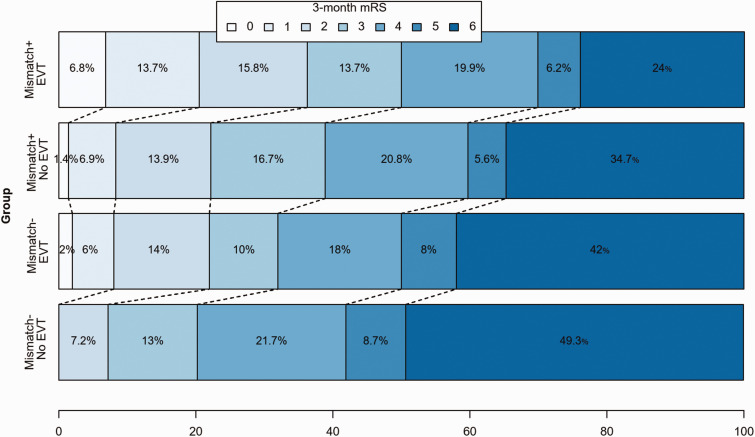
Distribution of scores for disability on the modified Rankin Scale (mRS) at 90 days among late-arriving patients who received (EVT) and did not receive endovascular treatment (No EVT), both in the subgroup of patients showing the clinical-ASPECTS mismatch (Mismatch+, top two bars) and in subgroup of patients without the clinical-ASPECTS mismatch (Mismatch*−*, bottom two bars). The scale ranges from 0 to 6, with higher scores indicating more severe disability. The numbers in the bars are percentages of patients who had each score.

Out of 2242 AIS patients who received EVT during the study period (2010–2018) in the two participating stroke centers, 482 (21.5%) were treated between 6 and 24 h from LPGH. Of these, 218 (9.7%) patients presented with admission NIHSS ≥10 and ICA/M1 occlusion, and were therefore included in the EVT-arm. To select the no-EVT arm, we screened 1675 AIS patients admitted in the late time window to Lausanne stroke center between 2005 and 2018, and we identified 119 (7.1%) patients who did not receive EVT and presented the clinico-radiological inclusion criteria. The flow chart for the selection of the study population is available in Figure 1S.

Therefore, the overall study cohort consisted of 337 AIS, with 172 patients from Lausanne and 165 patients from Bern stroke center. Their baseline demographics, clinic and radiological features are presented in Table 1. The median age was 73 (IQR = 61–82), with 52% females. The median NIHSS on admission was 18 (15–22) and median LPGH to hospital arrival was 9.2 (5.9–12.9) h. The median ASPECTS was 7 (5–9) and 24% of patients presented tandem occlusions.

**Table 1.** Baseline characteristics of the all included patients, and of the four groups of patients selected on the basis of clinical-ASPECTS mismatch (mismatch positive, mismatch negative) and late endovascular treatment (EVT, no EVT).

*Variables*	Total cohort (*N* = 337)	Mismatch + and EVT (*N* = 146)	Mismatch+ and no EVT (*N* = 50)	Mismatch*−* and EVT (*N* = 72)	Mismatch*−* and no EVT (*N* = 69)	*p-*value
Patients from Lausanne stroke center, *n* (%)	172 (51)	41 (28)	50 (100)	60 (83)	69 (100)	<0.001*
Age, median (IQR)	73 (61–82)	76 (62–84)	73 (58–83)	72 (56–81)	73 (63–83)	0.718
Sex (F), *n* (%)	176 (52)	80 (55)	28 (56)	31 (43)	37 (54)	0.371
Pre-stroke mRS<2, *n* (%)	268 (80)	129 (88)	29 (58)	65 (90)	45 (65)	<0.001*
*Vascular risk factors*						
Hypertension, *n* (%)	217 (64)	102 (70)	30 (60)	45 (63)	40 (58)	0.288
Diabetes, *n* (%)	59 (18)	30 (21)	14 (28)	7 (10)	8 (12)	0.024*
Dyslipidaemia, *n* (%)	218 (65)	91 (63)	36 (72)	48 (68)	43 (62)	0.619
Smoking, *n* (%)	81 (24)	33 (23)	11 (22)	18 (25)	19 (28)	0.887
*Treatment at stroke onset*						
Anticoagulation, *n* (%)	44 (13)	25 (17)	8 (16)	5 (7)	6 (9)	0.106
Antiplatelet, *n* (%)	102 (30)	41 (28)	15 (30)	23 (32)	23 (33)	0.879
Lipid lowering drugs, *n* (%)	82 (24)	35 (24)	14 (28)	20 (28)	13 (19)	0.579
NIHSS on admission	18 (15–22)	18 (14–22)	19 (15–23)	17 (15–20)	19 (16–24)	0.067
Glucose on admission, mmol/L	7.1 (6.2–8.6)	7.0 (6.1–8.5)	7.1 (6.2–8.9)	7.1 (6.0–8.4)	7.2 (6.5–8.6)	0.742
LPGH to presentation, min	549 (353–775)	492 (337–713)	614 (720–773)	424 (303–428)	741 (548–938)	<0.001*
Wake-up stroke, *n* (%)	169 (50)	62 (42)	34 (68)	32 (44)	41 (59)	0.004*
*Neuroimaging variables*						
MRI on admission, *n* (%)	96 (37)	59 (63)	0 (0)	37 (79)	0 (0)	<0.001*
Baseline ASPECTS	7 (5–9)	8 (7–9)	8 (7–9)	5 (3–6)	3 (2–5)	<0.001*
ICA occlusion, *n* (%)	149 (44)	54 (37)	25 (50)	32 (44)	38 (55)	0.070
Tandem occlusion, *n* (%)	81 (24)	31 (21)	16 (32)	14 (19)	20 (29)	0.249
IV thrombolysis, *n* (%)	50 (15)	31 (21)	3 (6)	14 (19)	2 (3)	0.001*
*EVT-related variables*						
LPGH to groin puncture, min	583 (423–789)	602 (432–819)	–	539 (403–689)	–	0.150
Groin puncture to recanalization, min	44 (28–70)	40 (27–67)	–	51 (33–80)	–	0.031*
General anesthesia, *n* (%)	185 (86)	123 (85)	–	62 (87)	–	0.623
Number of passes, *n*	1 (1–2)	1 (1–2)	–	1.5 (1–3)	–	0.568
Successful recanalization, *n* (%)	177 (81)	122 (84)	–	55 (76)	–	0.202

Note: Values are expressed as absolute counts and percentage for categorical variables, or medians and interquartile range (IQR) for continuous variables. *p*-values are given for the univariate difference between the four groups (or between the two EVT-groups for EVT-related variables).

mRS: modified Rankin Scale; NIHSS: National Institutes of Health Stroke Scale; LPGH: last proof of good health; ASPECTS: Alberta Stroke Program Early CT Score; ICA: internal carotid artery; EVT: endovascular treatment. *and bold characters denote significant results.

One hundred ninety-six (58.2%) patients presented a clinical-ASPECTS mismatch, of whom 146 (74.5%) received EVT. Among 141 (41.8%) patients without clinical-ASPECTS mismatch, EVT was performed in 72 (51.1%) patients.

The four groups of patients were well balanced for demographics, stroke severity, and vascular risk factors, with the exception of diabetes (higher in the mismatch positive – no EVT group) (Table 1). Patients who did not receive EVT showed a lower proportion of pre-stroke independency, longer delay from LPGH to hospital arrival, and higher frequency of wake-up strokes. Regarding neuroimaging features, more EVT patients were assessed by MRI. The median ASPECTS were lower in mismatch negative than in mismatch positive patients. Patients who did not receive EVT were also treated less frequently with IVT.

EVT-patients with the clinical-ASPECTS mismatch presented shorter delay between the groin puncture and the recanalization when compared to patients without. However, the median number of passes used and the rate of successful recanalization (i.e. mTICI ≥ 2b) were similar between the two groups.

The unadjusted ordinal shift analysis for three-month mRS showed that late EVT was associated with better outcome than absence of treatment in both patients with the clinical-ASPECTS mismatch (OR = 2.27; 95%CI = 1.21–4.16) and patients without the clinical-ASPECTS mismatch (OR = 2.34; 95% CI = 1.26–4.42). Also, among patients treated with late EVT, patients showing the clinical-ASPECTS mismatch presented better outcome compared to those without (OR = 1.74; 95%CI = 1.06–2.90) (Table 2). After adjusting for confounders, a favorable effect of late EVT on clinical outcome emerged in the mismatch positive group (OR = 2.83; 95%CI = 1.48–5.58), but no more in the mismatch negative group (OR = 1.32; 95%CI = 0.61–2.84). In this model, moreover, we found a near significant positive interaction between the clinical-ASPECTS mismatch and late EVT (*p* = 0.073) (Table 2).

**Table 2.** Results from the shift analyses for favorable outcome at three months (assessed with modified Rankin Scale) according to presence of clinical-ASPECTS mismatch and late endovascular treatment.

	Patients group	OR	95%CI	*p-*value
Unadjusted comparison				
EVT vs*.* No EVT	*Mismatch+*	2.27	1.21–4.16	**0.007** *
	*Mismatch−*	2.34	1.26–4.42	**0.008** *
Mismatch+ vs. Mismatch*−*	*EVT*	1.74	1.06–2.90	**0.030** *
	*No EVT*	1.68	0.83–3.38	0.147
*Interaction*^a^		1.10	0.47–2.58	0.832
Adjusted comparison^b^				
EVT vs. No EVT	*Mismatch+*	2.83	1.48–5.58	**0.002** *
	*Mismatch−*	1.32	0.61–2.84	0.484
Mismatch+ vs. Mismatch*−*	*EVT*	0.82	0.36–1.84	0.624
	*No EVT*	2.11	0.55–8.31	0.282
*Interaction*^a^		2.33	0.93–5.94	0.073

Note: Results are shown as odds ratios (OR) and their 95% confidence interval (CI), both for unadjusted and adjusted comparison.

* and bold characters means significant results.

a Refers to the interaction term between the presence of clinical-ASPECTS mismatch and late-EVT performed.

b Adjusted for: age, pre-stroke mRS, LPGH to presentation, NIHSS on admission, glucose on admission, baseline ASPECTS, IV thrombolysis.

Sensitivity analysis evaluating the primary outcome in the subgroup of patients assessed initially with CT only (*N* = 241) confirmed the direction and the magnitude of the ORs seen in the primary analysis, although the differences were no longer statistically significant (see Table 1S in the Supplementary material).

At 24 h, mismatch positive patients treated with late EVT showed a greater improvement in the NIHSS score compared to non-treated patients and mismatch negative patients (*p* < 0.001, Table 3). Compared to non-treated patients, patients receiving EVT showed higher rate of sICH (5% in mismatch positive patients, 13% in mismatch negative patients) (*p* = 0.017). At three months, only 25% of all patients achieved functional independency; this rate was significantly lower in non-treated patients without clinical-ASPECTS mismatch (7%, *p* < 0.001) (Figure 1). Also, there was a significant disproportion of mortality rates between the four groups (*p* = 0.002), with non-treated patients presenting higher proportions (42% in mismatch positive patients, 49% in mismatch negative patients) (Table 3 and Figure 1).

**Table 3.** Clinical outcome measures of the included patients, presented in the overall cohort, and in the four groups of interests.

*Variables*	Total cohort (*N* = 337)	Mismatch+ and EVT (*N* = 146)	Mismatch+ and no EVT (*N* = 50)	Mismatch- and EVT (*N* = 72)	Mismatch- and no EVT (*N* = 69)	*p-*value
Delta NIHSS at 24 h, median (IQR)	−1 (−7; 0)	−6 (−10; 0)	−1 (−2; 0)	0 (−6; 2)	0 (−1;1)	<0.001*
sICH, *n* (%)	18 (6)	7 (5)	2 (4)	9 (13)	0 (0)	0.017*
3-month mRS = 0–2, *n* (%)	85 (25)	53 (36)	11(22)	16 (22)	5 (7)	<0.001*
3-month mRS, median (IQR)	4 (2; 6)	4 (2; 6)	5 (3; 6)	4 (3; 6)	5 (4; 6)	0.001*
Death <3 months, *n* (%)	115 (34)	35 (24)	21 (42)	25 (35)	34 (49)	0.002*

NIHSS: National Institutes of Health Stroke Scale; delta NIHSS: NIHSS at 24 h – NIHSS on admission; mRS: modified Rankin Scale; mismatch: clinical-ASPECTS mismatch; EVT: endovascular treatment.

Note: UVA results from the comparison between groups are provided. *and bold characters mean significant results.

## Discussion

In our large, retrospective analysis of a two-centers cohort of late-arriving stroke patients undergoing EVT in routine clinical practice, we demonstrated a clinical benefit of the revascularization procedure in patients showing a mismatch between the stroke severity (assessed by NIHSS) and the amount of irreversibly damaged cerebral tissue (evaluated with the ASPECTS). After adjustment for multiple confounders, we found a near significant positive interaction between the treatment effect in the late time window and the selection of patients according to the proposed clinical-ASPECTS mismatch. In thus selected patients, treatment was also associated with early neurological improvement, similar risk of symptomatic hemorrhagic transformation, and lower mortality rate compared to non treated patients.

Real-world data regarding prevalence, treatments, and outcomes of LVO patients admitted in the extended time window fulfilling and not fulfilling DAWN and/or DEFUSE-3 criteria are very limited.^15,16^ These reports suggest that a less restrictive cut-off on infarct core volume was still able to select patients responding to late EVT. However, a simpler neuroimaging protocol for patients’ selection for mechanical thrombectomy beyond 6 h has not been tested yet, except in small case series.^8,17^ Our retrospective data show that a clinical-ASPECTS mismatch concept has potential value and safety to select patients for late thrombectomy, and that it might be not necessary to use more sophisticated perfusion imaging for this population.

Previous reports showed a moderate correlation between ASPECTS and core volumes on CTP.^18,19^ We previously demonstrated that ASPECTS assessment on NCCT seemed to be more accurate in later time windows and in patients with LVO, supporting the findings of our current analysis.^12^ This group of late-arriving patients might include patients with unknown onset or wake-up stroke, when the ASPECTS may be less reliable if the patient is actually early on after onset. Still, we did not find a significant difference in the strength of the ASPECTS-CTP-core correlation between patients with known and unknown stroke onset.^12^

Rates of good outcomes in our EVT cohort (36% in the mismatch positive patients and 22% in the mismatch negative patients) were lower compared to those found in the HERMES meta-analysis for EVT < 6 h,^20^ and in the DAWN (49%) and DEFUSE-3 (45%) trials. This could be explained by the real-life scenario of our study, including 20% of patients with significant pre-stroke disability (mRS ≥ 3). The non-treated group of our study also showed a rather poor natural course (with 16% patients achieving a good outcome), which was similar to the rates of functional independence in the control arm of DAWN (13%) and DEFUSE-3 (17%).

We found acceptable safety measures in our cohort of late EVT patient using less sophisticated imaging criteria: late EVT was not associated with higher risk of sICH in patients showing a clinical-ASPECTS mismatch (5% in treated patients vs. 4% in non-treated patients), with similar rates to those reported in late EVT trials. However, we found an increased risk of sICH for patients treated with late EVT not having the clinical-ASPECTS mismatch (13%). The median ASPECTS of these patients was low;^5^ therefore, this result is in line with the higher risk of intracranial hemorrhage after early EVT shown for patients with ASPECTS 0-4.^21^

Similarly, we found an increased mortality risk in treated patients not having the clinical-ASPECTS mismatch compared to treated patients showing the mismatch (35% vs. 24%, respectively); however, the EVT treatment was overall associated with a lower mortality risk when compared to non-treated patients (with 42% of deaths in the non-treated mismatch positive and 49% in the non-treated mismatch negative group, respectively). These percentages are in line with those observed in the DAWN trial (i.e. 25% in the thrombectomy group vs. 36% in the control group).

We acknowledge several limitations of our study. First, the nonrandomized and retrospective nature of the study might limit the generalization of the results. Second, it is possible that variables related to the decision to perform endovascular treatment have influenced our results; we have attempted to reduce this potential bias by including several of these variables in the adjustment for our final model. Third, the number of patients in each subgroup was likely insufficient to prove our hypotheses, leading to type II errors: our post hoc power calculation showed that more patients would be needed to prove the expected 18% improvement in the clinical outcome with 80% power. Therefore, the not quite significant *p*-value for interaction, as well as the absence of significance in the sensitivity analysis of CT-selected patients, might be explained by the low number of included patients. Fourth, the difference of neuroimaging modalities adopted in each center (with DWI- based ASPECTS mostly adopted in the EVT-group, and NCCT-based ASPECTS used for the non-treated group) might have influenced our results. Fifth, due to the long study period, especially for the non-treated arm, the outcomes measures might have been influenced by the improvement of revascularization treatments and general stroke care over time. Similarly, the differences in treatment selection approach between the two participating centers and over the study period might be a source of bias. Last, our goal was to assess the impact of patient selection for EVT on the real-world scenario of absent perfusion imaging where core volume cannot be precisely measured; therefore, perfusion-based analyses were not included in the current project.

## Conclusions

In our real-world analysis of consecutive stroke patients with proximal anterior circulation LVO, there seemed to be a more favorable outcome with late endovascular treatment in the setting of a mismatch between clinical severity and ASPECTS. This result could suggest a potential role of simpler neuroimaging protocols in late revascularization decisions, but confirmation by randomized controlled studies is needed.

## Supplemental Material

sj-pdf-1-wso-10.1177_17474930211009806 - Supplemental material for ASPECTS-based selection for late endovascular treatment: a retrospective two-site cohort study Supplemental material, sj-pdf-1-wso-10.1177_17474930211009806 for ASPECTS-based selection for late endovascular treatment: a retrospective two-site cohort study by Stefania Nannoni, Johannes Kaesmacher, Federico Ricciardi, Davide Strambo, Vincent Dunet, Steven Hajdu, Guillaume Saliou, Pasquale Mordasini, Arsany Hakim, Marcel Arnold, Jan Gralla, Urs Fischer and Patrik Michel in International Journal of Stroke
