# Supplementary material for: ASPECTS-based selection for late endovascular treatment: a retrospective two-site cohort study
Source: Int J Stroke. 2021 Apr 22;17(4):434–43. doi: 10.1177/17474930211009806 (PMC13029491; doi:10.1177/17474930211009806)
Supplement: sj-pdf-1-wso-10.1177_17474930211009806 - Supplemental material for ASPECTS-based selection for late endovascular treatment: a retrospective two-site cohort study [file sj-pdf-1-wso-10.1177_17474930211009806.pdf]

ASPECTS-based selection for late endovascular treatment:
a retrospective two-site cohort study

Supplementary materials

Supplementary Methods: Neuroimaging protocol; local endovascular treatment guidelines

Supplementary Table: Table 1S

Supplementary Figure: Figure 1S

Supplementary References (n): 9

Supplementary methods

Neuroimaging protocol

In *Lausanne stroke centre*, patients with suspected acute ischemic stroke (AIS) were examined with a multimodal neuroimaging protocol that included non-contrast CT scan (NCCT), CT angiography (CTA) and CT perfusion (CTP) until May 2018, and 3 Tesla magnetic resonance imaging (MRI) thereafter.

Cerebral CT was performed on a 64-multidetector CT scanner (LightSpeed VCT, GE Healthcare, Milwaukee, WI, USA). NCCT was acquired in axial mode using the following parameters: 120 kV peak tube voltage, 320 mA tube current, slice thickness 5 mm, 32 cm scan field of view (SFOV) and 512x512 matrix. Raw data were reconstructed in the axial plane using filtered-back-projection (FBP) until 2009, and adaptive statistical iterative reconstruction (ASiR) thereafter. The cervical and cerebral CTAs were acquired in helical scan mode from the aortic arch to the top of the frontal sinuses, according to the following parameters: 120 kV peak tube voltage, 150-260 mA tube current, 0.9:1 pitch, 0.625 mm slice thickness (1.25 mm before November 2005) and 512x512 matrix. Data acquisition was performed after intravenous injection of 50 ml of iodinated contrast material at a flow rate of 5 ml per second, with a delay according to the perfusion data.

Acute MRI was performed on a 3 Tesla scanner (Magnetom Vida®, Siemens Healthcare, Erlangen, Germany). The following sequences were acquired consecutively: sagittal gradient echo T1 (TR 400 ms, TE 2.46 ms, flip angle 70º, 3 mm slice thickness, matrix 320 x 300), axial diffusion (TR 3000 ms, TE 80 ms, 3 mm slice thickness, 52 slices, matrix 148x148, 20 directions, b-value 0 and 1000 s/mm2), axial 2D fluid attenuated inversion recovery (FLAIR, TR 9000 ms, TE 87 ms, TI 2500 ms, flip angle 150º, 3 mm slice thickness, matrix 640x480), axial gradient echo T2 (TR 1070 ms, TE 19.8 ms, flip angle 20º, 3 mm slice thickness, matrix 320 x 272), time-of-flight angiography (TR 22 ms, TE 3.6 ms, flip angle 20º, 0.5 mm slice

thickness, matrix 896 x 720), cervical T1 SPACE fat sat coronal (TR 723 ms, TE 20 ms, 0.9 mm slice thickness, matrix 256 x 220), cervical MRA (TR 3.49 ms, TE 1.23 ms, TTC 5.0 sec, 0.8 mm slice thickness, matrix 448 x 336), post-contrast axial gradient echo T1 (TR 400 ms, TE 2.61 ms, 3 mm slice thickness, matrix 448 x 380) and perfusion weighted imaging (TR 2430 ms, TE 25 ms, 3 mm slice thickness, 40 slices, matrix 140 x 140, resolution time 1.5s over 90 sec). Apparent diffusion coefficient (ADC) map was also generated from diffusion weighted imaging sequence.

We had previously assessed interrater variability between the vascular neurologist responsible for this project (PM) and a senior neuroradiologist using the Cohen k statistic on 100 consecutive acute CT scans with anterior circulation occlusive stroke, finding an almost perfect agreement for visual ASPECTS scoring (k = 0.82).

NCCT/DWI images were reviewed for ASPECTS retrospectively by an experienced vascular neurologist (PM). For DWI-based ASPECTS, one point was added before analysis because of its higher sensitivity than CT-ASPECTS (1). This person compared his value to the assessment established by the radiologist in the acute phase and appearing in the official (clinical) radiology report. In cases of disagreement between the two ASPECTS values, the case was discussed at the weekly joint neuroradiology meetings to reach a consensus.

In *Bern stroke centre*, admission imaging was performed either with 1.5T/3T MRI (Magnetom Avanto or Magnetom Verio, Siemens Healthcare, Erlangen, Germany) or with 128-multidetector CT (SOMATOM Definition Edge, Siemens Healthcare, Erlangen, Germany). For MRI, the following sequences were included in the acute stroke protocol: DWI, FLAIR, SWI, TOF, PWI, post contrast T1, CE MRA of the cervical vessels. Scanner parameters for axial DWI were: TR 3000ms, TE 89ms, number of averages 4, FOV 230x230, voxel 1.2x1.2x5mm, slice thickness 5mm, matrix 192x192 for 1.5T; TR 3500ms, TE 89ms, number of averages 4, FOV 230x230, voxel 1.8x1.8x5mm, slice thickness 4mm, matrix 128x128, for

3.0T. Yielding isotropic b0 and b1000 as well as apparent diffusion coefficient (ADC) maps were calculated automatically. Scanner parameters for magnetic resonance perfusion were as follows: repetition time: 1410 ms, echo time: 30 ms, field of view: 230×230 mm, voxel size: 1.8×1.8×5.0 mm, slice thickness: 5.0 mm, number of slices: 19, and flip angle: 90°. Perfusion maps were generated using the Olea Sphere Software environment (Olea Sphere v2.3; Olea Medical, La Ciotat, France). CT included thin slice non-contrast CT (slice thickness: 1 mm), perfusion CT (automatically processed by multimodality workstation, syngo.via, Siemens Healthcare, Erlangen, Germany), and CT angiography in early arterial (bolus triggered, slice thickness: 0.6 mm) and late venous phase (75 seconds delay, slice thickness: 1 mm).

Local endovascular treatment (EVT) guidelines

EVT guidelines have evolved during the study period. *In Lausanne stroke centre*, EVT was performed till 2014 if treatment could be initiated within 6 hours, NIHSS was ≥6, CTA disclosed a proximal intracranial vessel occlusion (2) and CT-perfusion (CTP) showed >50% of penumbra in anterior circulation strokes. Later arriving patients and unknown onset patients were treated in selected cases if CTP showed >50% penumbra and informed consent was available. Since 10/2014, CTP criteria were replaced by ASPECTS ≥5 and lower NIHSS limit was replaced by the presence of a disabling deficit (3), similar to the European criteria (4). Since May 2017, patients were treated with the same criteria but up to 8 hours (5). After 8 hours, treatment was offered with modified DAWN criteria, i.e. in the presence of an NIHSS ≥10 and ASPECTS ≥7, or if stroke was disabling, NIHSS was 1-10, and ASPECTS was ≥8 (6, 7). Since January 2018, late treatment was alternatively based on any NIHSS, core <70 mL and mismatch ratio ((penumbra + core)/core) >1.8, according to DEFUSE-3 criteria, and in accordance with European (8) and American recommendations (9).

In *Bern stroke centre*, clinical eligibility criteria for EVT were NIHSS score ≥4 or NIHSS <4 with relevant deficits (a.g. aphasia, distal paresis). Treatment was considered on individual case basis in patients with NIHSS <4 without relevant deficits and/or rapidly improving symptoms but persistent vessel occlusion. EVT was performed up to 8h in ICA, carotid-T, M1 and M2 occlusion without further imaging criteria. From 8h-24h since LPGH, EVT was considered in case of clinical mismatch (locally defined as NIHSS≤10 and core <1/3 of the MCA territory). EVT was considered despite core >1/3 of MCA territory in younger patients. An overview of the local guidelines can be found online: http://www.neurologie.insel.ch/fileadmin/neurologie/neurologie_users/Unser_Angebot/Dokumente/Stroke-Guidelines-07-19-english.pdf

Table 1S. Results from a sensitivity analysis for favourable outcome at 3 months (assessed using shift in the modified Rankin Scale) according to presence of clinical-ASPECTS mismatch and late endovascular treatment (EVT), performed in the subgroup of patients assessed with CT on admission (N=241). Results are shown as odds ratios (OR) and their 95% confidence interval (CI).

Adjusted analysis*	**Patients group**	**OR**	**95%CI**	***p*-value**
**EVT *vs* No EVT**	*Mismatch+*	2.34	0.78-7.09	0.127
	Mismatch-	1.47	0.28-7.50	0.640
**Mismatch+ *vs* Mismatch-**	*EVT*	0.60	0.11-3.64	0.561
	No EVT	1.99	0.52-7.84	0.318
Interaction°		1.38	0.30-6.60	0.680

Legend:

*Adjusted for: age, pre-stroke mRS, LPGH to presentation, NIHSS on admission, glucose on admission, baseline ASPECTS, IV thrombolysis.

°Refers to the interaction term between the presence of clinical-ASPECTS mismatch and late-EVT performed.

Figure 1S. Flow-chart of the study population, showing the inclusion of patients treated with late-EVT (from Lausanne and Bern stroke centres) and the selection of control patients who did not received late-EVT (from Lausanne stroke centre only).

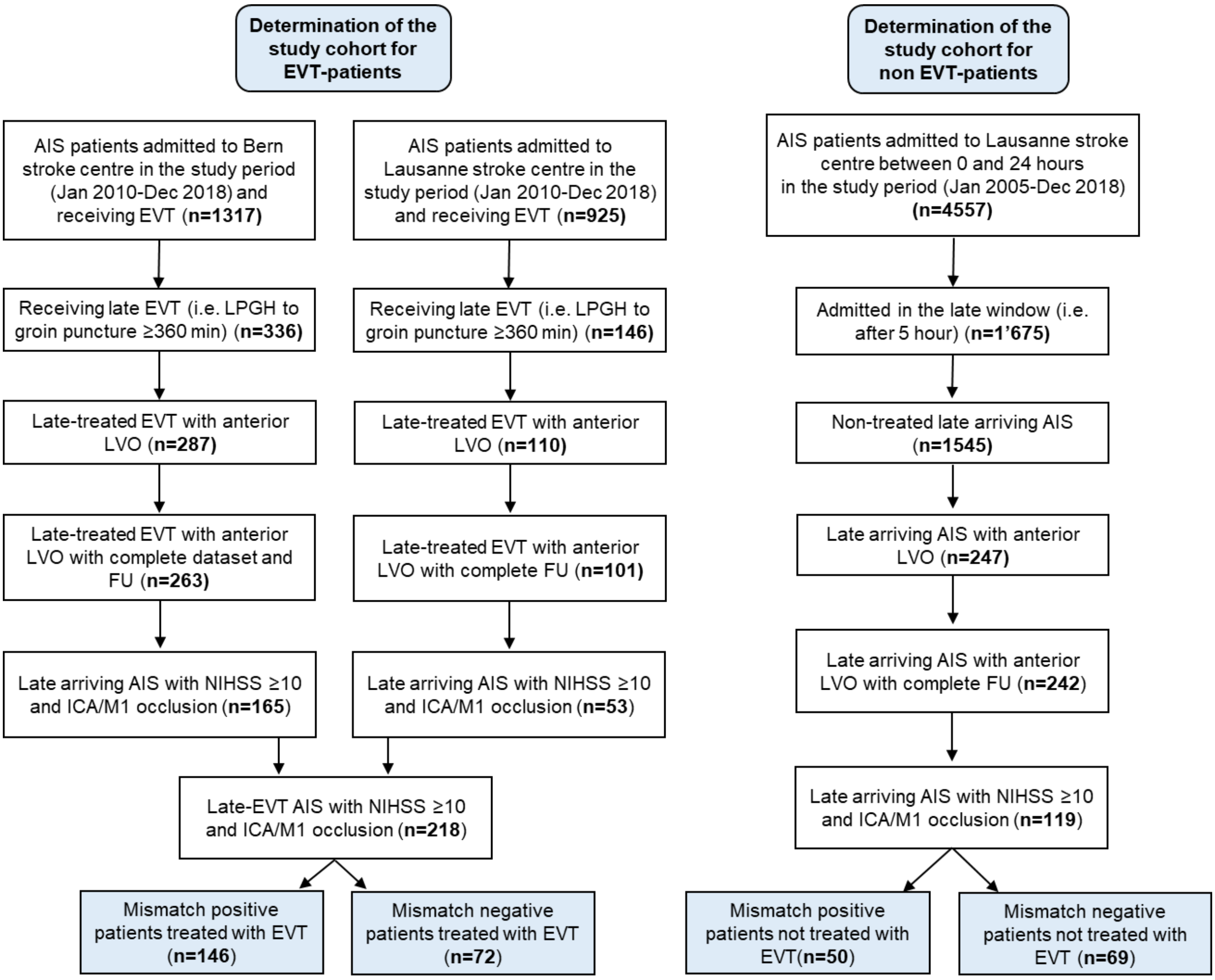

Legend: AIS= acute ischemic stroke; ASPECTS= Alberta Stroke Program Early CT Score; EVT=endovascular treatment; FU= follow-up; ICA=internal carotid artery; LPGH= last proof of good health; LVO=large vessel occlusion; mismatch positive/negative= presence/absence of clinical-ASPECTS mismatch; NIHSS= National Institutes of Health Stroke Scale.

Supplementary references

1. Nezu T, Koga M, Nakagawara J, Shiokawa Y, Yamagami H, Furui E, et al. Early ischemic change on CT versus diffusion-weighted imaging for patients with stroke receiving intravenous recombinant tissue-type plasminogen activator therapy: stroke acute management with urgent risk-factor assessment and improvement (SAMURAI) rt-PA registry. Stroke. 2011;42(8):2196-200.
2. Michel P, Engelter S, Arnold M, Hungerbühler H, Nedeltchev K, Georgiadis D, et al. Thrombolyse de l'attaque cérébrale ischémique : Recommandations actualisées. Swiss Medical Forum. 2009;9(49):892-4.
3. Berkhemer OA, Fransen PS, Beumer D, van den Berg LA, Lingsma HF, Yoo AJ, et al. A randomized trial of intraarterial treatment for acute ischemic stroke. N Engl J Med. 2015;372(1):11-20.
4. Fiehler J, Cognard C, Gallitelli M, Jansen O, Kobayashi A, Mattle HP, et al. European Recommendations on Organisation of Interventional Care in Acute Stroke (EROICAS). International journal of stroke : official journal of the International Stroke Society. 2016;11(6):701-16.
5. Saver JL, Goyal M, van der Lugt A, Menon BK, Majoie CB, Dippel DW, et al. Time to Treatment With Endovascular Thrombectomy and Outcomes From Ischemic Stroke: A Meta-analysis. Jama. 2016;316(12):1279-88.
6. Nogueira RG, Jadhav AP, Haussen DC, Bonafe A, Budzik RF, Bhuva P, et al. Thrombectomy 6 to 24 Hours after Stroke with a Mismatch between Deficit and Infarct (DAWN). N Engl J Med. 2018;378(1):11-21.
7. Nannoni S, Ricciardi, F., Strambo, D., Sirimarco, G., Marto, J. P., Bartolini, B., Wintermark, M., Dunet, V., Patrik, M. Reproducing DAWN rersults in real life based on clinical-ASPECTS mismatch: an explanatory analysis [poster]. European Stroke Organisation Conference 2019, Milan. Eur Stroke J. 2019.
8. Turc G, Bhogal P, Fischer U, Khatri P, Lobotesis K, Mazighi M, et al. European Stroke Organisation (ESO) - European Society for Minimally Invasive Neurological Therapy (ESMINT) Guidelines on Mechanical Thrombectomy in Acute Ischaemic StrokeEndorsed by Stroke Alliance for Europe (SAFE). Eur Stroke J. 2019;4(1):6-12.
9. Powers WJ, Rabinstein AA, Ackerson T, Adeoye OM, Bambakidis NC, Becker K, et al. Guidelines for the Early Management of Patients With Acute Ischemic Stroke: 2019 Update to the 2018 Guidelines for the Early Management of Acute Ischemic Stroke: A Guideline for Healthcare Professionals From the American Heart Association/American Stroke Association. Stroke. 2019;50(12):e344-e418.
